# Effects of silk peptides administration on fat utilization over a whole day in mice

**DOI:** 10.20463/jenb.2016.0055

**Published:** 2016-12-31

**Authors:** Jisu Kim, Jonghoon Park, Bokyung Kim, Chi-Ho Lee, Kiwon Lim, Heajung Suh

**Affiliations:** 1 Physical Activity and Performance Institute (PAPI), Konkuk University, Seoul Republic of Korea; 2 Department of Physical Education, Korea University, Seoul Republic of Korea; 3 Department of Medicine, Konkuk University, Chungju Republic of Korea; 4 Department of Food Science and Biotechnology of Animal Resources, Konkuk University, Seoul Republic of Korea; 5 Department of Physical Education, Konkuk University, Seoul Republic of Korea

**Keywords:** Silk peptides, RMR, Energy expenditure, FAT/CD36

## Abstract

**[Purpose]:**

The aim of the present study was to investigate the effects of treatment with silk peptides (SP) on resting energy expenditure over a 24-h period and clarify the molecular mechanism underlying its enhancement of fat utilization in mouse skeletal muscles.

**[Methods]:**

Sixteen male ICR mice (6-weekold) were divided into two groups and treated with distilled water and SP (CON and SP treatment groups, respectively). SP were dissolved in distilled water and administered to the SP group at 800 mg/kg while the CON group was administered distilled water orally daily for 2 weeks. Resting metabolic rate (RMR) was measured before and after SP ingestion for 2 weeks using an open circuit calorimetry system. After the treatments, we collected blood and skeletal muscle samples from the mice.

**[Results]:**

Final body weight and body weight gain showed no significant difference in the CON and SP groups. Conversely, food intake was significantly lower in the SP group than it was in the CON group. The comparison of the respiratory exchange rate (RER) at various time points after the 2-week treatment revealed that it was significantly lower in the SP group than it was in the CON group at 9, 15, 16, and 18-22 h (Figure 3 B). The sum of the RER over 24-h was lower in the SP group than it was in the CON group, but the difference was not statistically significant. The protein levels of fatty acid translocase (FAT)/ cluster of differentiation 36 (CD36) were approximately 12% higher in the SP group than they were in the CON group but that of carnitine palmitoyltransferase I (CPTІ) was not significantly different between the two groups.

**[Conclusion]:**

These results suggest that treatment with SP 800 mg/kg for 2 weeks may promote fat utilization during physical activity, but not the entire day. In addition, SP treatment effectively enhanced FAT/CD36 protein level in skeletal muscle. In future investigations, it would be necessary to elucidate the effects of long-term SP intake on the resting metabolism of both animals and humans.

## INTRODUCTION

Silk peptides (SP) are known to include biopolymers from the cocoons produced by silkworms. The SP component is similar to proteins such as collagen, elastin, keratin, fibroin, and sporgin, and is an important ingredient of cocoons^1, 2^. In additional, SP has been ingested as a health functional food in Asian countries3. Numerous studies on the health benefits of SP intake have been reported^4-7^. SP not only regulate blood glucose levels and hyperlipidemia, but they also decrease blood triglycerides and low-density lipoprotein (LDL)-cholesterol^8, 9^.

Recently, SP (100 mg/kg) treatment was reported to decrease the plasma levels of total cholesterol, LDL-cholesterol, and the atherogenic index by 11%, 27%, and 26%, respectively compared to the values reported for the control group. Additionally, this study suggests that SP treatment improved insulin resistance by inducing β-cell activity in the pancreatic islets in mice^9^. Shin SH et al. (2009)^10^ reported that high-dose (800 mg/kg) SP treatment increased anti-fatigue by preventing glycogen depletion. In another study, dietary SP (0.5% and 0.8%) treatment reduced body weight and increased serum leptin levels^11^. Moreover, a 2-month intake of SP with green tea decreased body fat, total cholesterol, and LDL-cholesterol in human subjects^12^. The results of these studies suggest that SP intake may be related to enhanced fat metabolism and have an anti-obesity effect. However, the studies mentioned above only measured the blood profiles or hormones in tissues.

Recently, we reported that SP (800 mg/kg) treatment with endurance training (approximately 70-75% maximal oxygen uptake) improved fat oxidation at rest and during exercise considerably more than the control treatment with endurance training^13, 14^. However, it is still unclear whether SP administration would only affect whole-body fat utilization and alter gene expression related to fat metabolism in muscle tissues. Therefore, the aim of the present study was to investigate the effects of SP treatment on resting energy expenditure over 24 h and clarify the molecular mechanism underlying the enhancement of fat utilization in mouse skeletal muscles.

## METHODS

### Animal

Sixteen male ICR-mice (6-week-old) were obtained from Orient Bio Inc. (Seongnam) and were allowed to adapt to the laboratory housing conditions for 1 week. At the age of 7 weeks, the mice were randomized to the control (CON, distilled water) and SP treatment (SP, 800 mg/ kg) groups. Food intake and body weight were measured daily for 2 weeks, and the day after the experimental protocol, the mice were euthanized, and blood and skeletal muscle tissue samples were collected for protein expression analysis using western blotting.

### SP preparation and administration

The SP were obtained from Worldway Co., Ltd., (Jeonui, Korea) and their molecular weight was approximately 150-350 D with an average of approximately 250 D. The SP were dissolved in distilled water and administered to the SP group at 800 mg/kg, while the CON group was orally administered distilled water daily for 2 weeks.

### Resting metabolic rate (RMR) analysis

The resting metabolic rate (RMR) was measured before and after SP ingestion for 2 weeks. Two hours before the measurement, the mice were placed in a metabolic chamber with a volume of approximately 1-L to reduce stress. The flow rate was kept constant at 1.2 L/min and measured for 24 h^15^. An unpurified commercial diet and water were given freely to the mice. The RMR was measured using an open-circuit device based on methods reported in previous studies^15, 16^.

### Blood analysis

Blood samples were collected in ethylenediaminetetraacetic acid (EDTA) tubes (BD vacutainer K2 EDTA 10.8 mg, plus blood collection tubes, USA), and centrifuged at 3,000 rpm at 4°C for 15 min. The plasma glucose level was measured as follows using the glucose oxidase method. Briefly, 1 ml reaction buffer was added to the supernatant (10 μL) obtained above, and the glucose level was determined using a glucose test kit (Asan Pharmaceutical Co., Ltd.). The absorbance of the reaction mixture was measured at 505 nm using a spectrophotometer (Multiskan Go microplate reader, ThermoFisher, CA, USA).

The plasma insulin was measured using Morinaga ultra-sensitive mouse/rat insulin enzyme-linked immunosorbent assay (ELISA) kits using the guinea pig anti-insulin antibody reaction. Briefly, the sample was bound to the guinea pig anti-insulin antibody coated on the microplate well, and the unbound material was removed using a washing buffer. The horseradish peroxidase (HRP)-conjugated anti-insulin antibody was then bound to the guinea pig anti-insulin antibody mouse insulin complex immobilized on the microplate well. Next, the excess RP-conjugate was removed using a washing buffer while the bound form on the microplate well was detected by adding 3,3’, 5,5’-tetramethylbenzidine (TMB) substrate solution. The samples were subsequently measured at 450 and 630 nm using a spectrophotometer (Multiskan Go microplate reader).

The plasma free fatty acids (FFAs) were measured using a nonesterified FA (NEFA) kit (Waco Pure Chemical Industries, Osaka, Japan). Briefly, reagent A was prepared by mixing 1 vial each of chromogen reagent A and solvent A (10 mL each) and reagent B was prepared by mixing 1 vial each of chromogen reagent B and solvent B (20 mL each). Then, reagent A (80 μL) was added to the sample (4 μL), mixed thoroughly, incubated at 37°C for 10 min, and the mixture was added to color reagent B (160 μL), followed by thorough mixing and incubation at 37°C for 10 min. The reaction mixture was subsequently measured at 550 nm using a spectrophotometer (Multiskan Go microplate reader).

### Protein extraction and western blot analysis

The muscle (gastrocnemius) tissue samples were homogenized using a mortar and TissueRuptor (QIAGEN, Germany) prior to western blot analysis. The muscle tissue (30 mg) was homogenized in 800 μL radioimmunoprecipitation assay (RIPA) lysis buffer (EzRIPA lysis, ATTO Biotechnology, Sungnam, Korea). The muscle lysates were mixed using a rotator for 2 h at 4°C and then centrifuged at 12,000 rpm at 4°C for 15 min. The protein concentration of the supernatant was determined using a GenDEPOT protein assay plus reagent kit (Gen-Depot Laboratories, USA) using bovine serum albumin (BSA) as the standard.

Total protein (25 μg/lane) was separated using 12% sodium dodecyl sulfate (SDS)-polyacrylamide gel electrophoresis (PAGE) at 80-110 v for 150 min and then transferred to a polyvinylidene difluoride (PVDF) membrane (Millipore, Billerica, MA, USA) at 100 v for 2 h. The membrane was blocked for 1 h at room temperature with phosphate-buffered saline (HyClone Laboratories, USA) containing 5% skim milk (Difco, USA) and then washed thrice (5, 5, and 15 min) with PBS plus 0.1% Tween 20 (PBS-T) buffer. After an overnight incubation at 4°C with primary antibodies against fatty acid translocase (FAT)/ cluster of differentiation 36 (CD36) and carnitine palmitoyltransferase I (CPTІ, Santa Cruz Biotechnology, USA), the membranes were washed with PBS-T and incubated with an HRP-conjugated secondary antibody for 1 h at room temperature.

Immunodetection was carried out using an enhanced chemiluminescence (ECL) detection reagent (Amersham Biosciences, Uppsala, Sweden). All figures showing the results of the quantitative analyses using the Image J program (National Institutes of Health, NIH, Bethesda, MD, USA) include data from at least three independent experiments.

### Statistical analysis

The data are presented as the mean ± standard deviation (SD) and all statistical analyses were performed using the statistical package for the social sciences (SPSS) version 19.0 software (SPSS, Inc., Chicago, IL, USA). Oxygen (O_2_) uptake, carbon dioxide (CO_2_) production, respiratory exchange rate (RER), food intake, and body weight were analyzed using a two-way repeated measures analysis of variance (ANOVA). These results were further evaluated using an unpaired t-test to compare the time points between groups. The blood profiles were analyzed using an unpaired t-test and differences were considered significant at P < 0.05.

## RESULTS

### Body weight, food intake, and adipose tissue

Table 1 shows the change in body weight, food intake, and adipose tissue weight in the CON and SP groups. The final body weight (36.8 ± 2.3 and 37.12 ± 1.3 g) and body weight gain (1.55 ± 1.0 and 1.9 ± 1.5 g) showed no significant difference between the CON and SP groups, respectively. However, the food intake was significantly lower in the SP group than it was in the CON group. There was also no significant difference in the food efficiency ratio and abdominal tissues between both groups.

**Table 1.** Change in body weight, food intake, and adipose tissue in control (CON) and silk peptide (SP) groups

	CON	SP
Initial body weight (g)	35.3 ± 1.7	35.2 ± 1.4
Final body weight (g)	36.8 ± 2.3	37.12 ± 1.3
Body weight gain (g)	1.55 ± 1.0	1.9 ± 1.5
Food intake (g/day)	6.96 ± 1.7	5.36 ± 0.8***
Food efficiency ratio	0.21 ± 0.1	0.34 ± 0.2
Abdominal tissue (g)		
Epididymal	0.71 ± 0.1	0.78 ± 0.2
Perirenal	0.3 ± 0.0	0.4 ± 0.1
Mesenteric	0.67 ± 0.0	0.71 ± 0.0
Total adipose tissue	1.70 ± 0.2	1.87 ± 0.3

**Change in body weight, food intake, and adipose tissue weight in CON and SP groups**. Values are means ± standard deviations (SD)

***P < 0.001 vs CON group

### RMR

**Figure 1.**
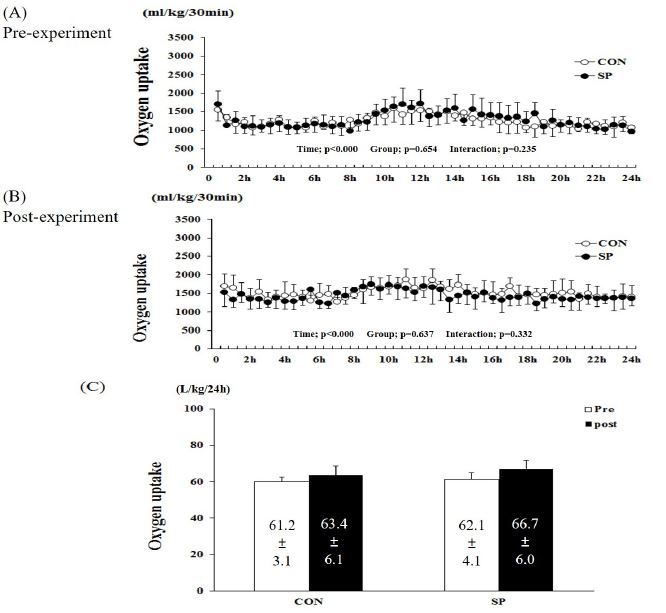
**Changes in oxygen (O2) uptake during (A) pre- and (B) post-experimental periods.** (C) Sum of the O2 uptake over 24 h in SP and CON groups. SP, silk peptide group (800 mg/kg); CON, control group. Values are means ± standard deviations (SD, n = 16).

The two-way repeated measures ANOVA of the O_2_ uptake showed a significant time-related effect (P < 0.001, pre- and post-experimental). However, no group (P = 0.654 and 637 in the pre- and post-experimental periods, respectively) and group-by-time interactions were observed (P = 0.235 and 0.332, pre- and post-experimental, respectively, Figure 1). The sum of the O_2_ uptake over 24-h in the pre-CON, -SP, and post-CON, and -SP groups was 61.2 ± 3.1, 63.4 ± 6.1, 62.1 ± 4.1, and 66.7 ± 6.0 L/kg/24 h, respectively (Figure 1C). There was no difference between the pre- and post-experimental conditions in the two groups.

**Figure 2.**
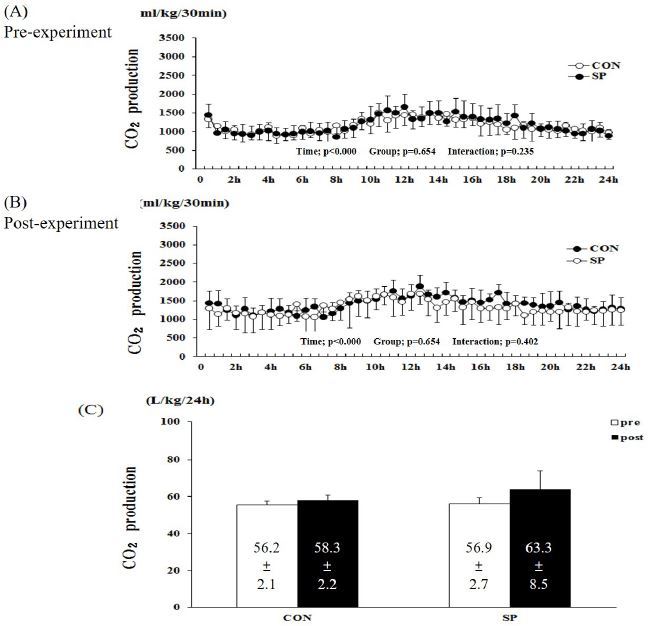
**Changes in carbon dioxide (CO2) production during (A) pre- and (B) post-experimental periods.** (C) Sum of CO2 production over 24 h in SP and CON groups. SP, silk peptide group (800 mg/kg); CON, control group. Values are means ± standard deviations (SD, n = 16).

The two-way repeated measures ANOVA for CO2 production showed a significant time-related effect (P < 0.001, pre- and post-experiment), but not group (P = 0.777 and 654, pre- and post-experiment, respectively) and group-by-time interactions (P = 0.402 and 0.402, pre- and post-experiment, respectively, Figure 2). The sum of the CO2 production over 24 h in the pre-CON, -SP, postCON, and -SP groups was 56.2 ± 2.1, 58.3 ± 2.2, 56.9 ± 2.7, and 63.3 ± 8.5 L/kg/24 h, respectively (Figure 2C). There was no difference between the pre- and post-experimental conditions in the two groups.

**Figure 3.**
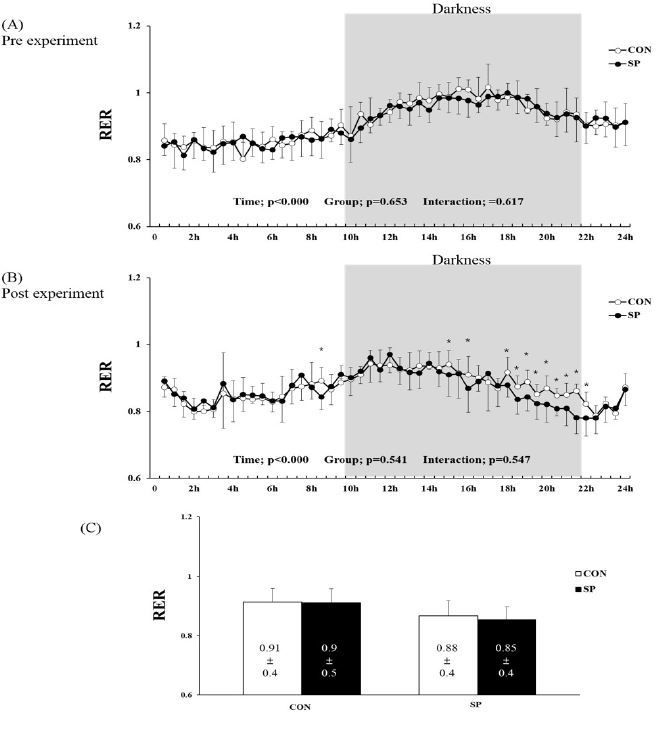
**Changes in respiratory exchange rate (RER) during (A) pre- and (B) post-experimental periods.** (C) Mean RER over 24 h in SP and CON groups. SP, silk peptide group (800 mg/kg); CON, control group. *P < 0.05 for CON vs. SP group. Values are means ± standard deviations (SD, n = 16).

The RER, calculated as the ratio of the O_2_ and CO_2_ produced over 24 h, was observed to change over time under the pre- and post-treatments (Figures 3A and 5B, before and after treatment, respectively) and after the experiment (P < 0.000, Figure 3A and 3B). However, no group-by-time interaction and group effect were observed in the pre- and post-experimental periods (Figure 3A and 3B). Furthermore, the difference in the RER at the various time points evaluated after the 2-week experiment was significantly lower in the SP than it was in CON group at 9, 15, 16, and 18-22 h (Figure 3B). The sum of the RER over 24-h tended to be lower (6 and 4%) in the SP group than it was in the pre-SP and post-CON groups, respectively (Figure 3C). However, there was no significant difference between the two groups.

### Blood analysis

**Figure 4.**
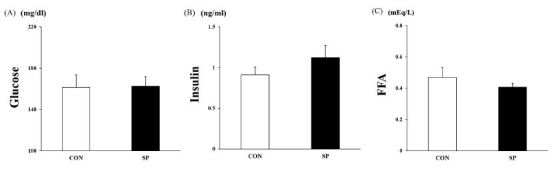
**Changes in plasma glucose, insulin, and free fatty acid (FFA)** levels Blood analyses were performed after 2-week silk peptide (SP) treatment to measure plasma (A) glucose, (B) insulin, and (C) FFAs. Values are means ± standard deviations (SD, n = 16).

The plasma glucose, insulin, and FFAs levels showed no significant difference between the two groups (Figure 4).

### Expression of FAT/CD36 and CPTІ in skeletal muscle

**Figure 5.**
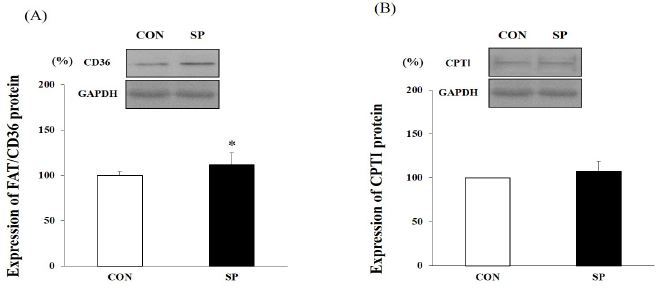
**Expression levels of fatty acid translocase (FAT)/cluster of differentiation 36 (CD36) and carnitine palmitoyltransferase I (CPTІ) in skeletal muscle analyzed using western blotting** Results are expressed as relative activity during and after 2-week silk peptides (SP) intake in SP and untreated control (CON) group. Expression of (A) FAT/CD36 and (B) CPTІ proteins. Bars represent means ± standard deviation (SD); *P < 0.05 vs. CON group.

Western blot analysis was performed using the protein obtained from the mouse skeletal muscle (gastrocnemius) samples. The FAT/CD36 protein level in the SP group was approximately 12% higher than it was in the CON group (P < 0.05) but the CPTІ protein level did not differ significantly between the two groups (Figure 5).

## DISCUSSION

We found that a 2-week SP (800 mg/kg) treatment did not change the O_2_ uptake and CO_2_ production for 24 h. The mean RER over a 24-h period was 6% and 4% higher in the SP group than it was in the CON group but the difference was not statistically significant. Particularly, SP (800 mg/kg) treatment was shown to decrease the RER (i.e., increased fat utilization) at some time points between 10-22 h during the dark phase when rodents are physically active. We also found that the FAT/CD36 protein level in the SP group was significantly higher than it was in the CON group.

We previously reported that the group exposed to a 2-week treatment with SP (800 mg/kg with endurance training) exhibited a higher increase in O_2_ uptake and fat oxidation at the resting metabolic rate than the exercise-only group13. However, in the present study, the SP treatment alone did not significantly increase the energy expenditure or fat oxidation for 24 h, whereas the RER appeared to decrease during the dark period when rodents are active. Several studies have indicated that SP intake elevated thermogenesis reactions during physical activities, resulting in a reduction of body weight and fat^12, 17^. These studies evaluated the effect of a long experimental period (42, 44, and 56 days) and SP administration and used a longer experimental duration that this present study did. Therefore, we cautiously assumed that treatment with 800 mg/kg SP (although considered an optimal dose) for 2 weeks was not adequate to increase the sum of the fat utilization during a 24-h period, but may be effective in combination with endurance training.

In the present study, the FAT/CD36 protein levels in the skeletal muscle were higher in the SP group than they were in the CON group. Recently, FAT/CD36 was reported to be a key component of the molecular machinery required for regulating skeletal muscle fuel selection at various lipolysis states^18^. In particular, it has been reported that FAT/CD36 markedly increases during the lipolysis process. In the present study, our data revealed that the SP (800 mg/kg) group appeared to spend more fat energy, especially during the period of darkness (physically active time) than the CON group did. At this time, we observed no change in the blood glucose, FFA, and insulin levels.

However, a limitation of this study is that we did not measure the physical activity of the mice over an entire day. However, we confirmed that 2 weeks of SP intake increased FAT/CD36 protein expression in the skeletal muscle and, therefore, we believe that the effect of SP intake on RER during the dark phase was due to the increased fat utilization in the skeletal muscle and not increased physical activity induced by SP intake.

In conclusion, these results suggest that treatment with 800 mg/kg for 2 weeks may promote fat utilization during physically activity periods, but not during an entire day. In addition, the SP treatment effectively enhanced FAT/ CD36 protein levels in the skeletal muscle. In future investigations, it would be necessary to elucidate the effects of long-term intake of SP on the resting metabolism of both animals and humans.

## COMPETING INTERRESTS

The authors declare that they have no competing interests.

## References

[1] Jung EY, Lee HS, Lee HJ, Kim JM, Lee KW, Suh HJ (2010). Feeding silk protein hydrolysates to C57BL/KsJ-db/db mice improves blood glucose and lipid profiles. *Nutr Res*.

[2] Ahmad R, Kamra A, Hasnain SE (2004). Fibroin silk proteins from the nonmulberry silkworm Philosamia ricini are biochemically and immunochemically distinct from those of the mulberry silkworm Bombyx mori. *DNA Cell Bio*.

[3] Lee SH, Park GY, Bae DK, Yang YH (2012). Silk and silkworm pupa peptides suppress adipogenesis in preadipocytes and fat accumulation in rats fed a high-fat diet. *Eur J Nutr*.

[4] Park DS, Lee SH, Choi YJ, Bae DK, Yang YH, Goeun Y, Kim TK, Yeon SH, Hwang SY, Joo SS, Kim YB (2011). Improving effect of silk peptides on the cognitive function of rats with aging brain facilitated by d-galactose. *Biomol Ther*.

[5] Kim TK, Park DS, Yeon SH, Lee SH, Choi YJ, Bae DK, Yang YH, Yang GE, Joo SS, Lim WT, Lee JY, Lee JS, Jeong HS, Hwang SY (2011). Tyrosine-fortified silk amino acids improve physical function of parkinson’s disease rats. *Food Sci Bioteclnol*.

[6] Kim DW, Hwang HS, Kim DS, Sheen SH, Heo DH, Hwang G, Kang SH, Kweon H, Jo YY, Kang SW, Lee KG, Park J, Eum WS, Cho YJ, Choi SY (2012). Enhancement of anti-inflammatory activity of PEP-1-FK506 binding protein by silk fibroin peptide. *J Microbiol Biotechnol*.

[7] Noriaki N, Takatoshi M, Yoshimasa I, Norio O, Masahiro S (2009). Enhancing effects of sericin on corneal wound healing in otsuka longevans thkushima fatty rats as a model of human type 2 diabetes. *Biol Pharm Bull*.

[8] Hwang EH, Kang BG, Kim BY, Lee HJ (2001). Protein quality evaluation and effect of plasma lipid contents of acid hydrolysates of cocoon in rats fed by high cholesterol, high triglyceride and high sucrose diet. *J Kor Soc Food Sci Nutr*.

[9] Jung EY, Lee HS, Lee YJ, Kim JM, Lee K, Suh HJ (2010). Feeding silk protein hydrolysates to C57BL/KsJ-db/db mice improves blood glucose and lipid profile. *Nutr Res*.

[10] Shin SH, Park DS, Yeon SH, Jeon JH, Kim TK, Joo SS, Lim WT, Lee JY, Kim YB (2009). Stamina-enhancing effects of silk amino acid preparations in mice. *Lab Anim Res*.

[11] Lee YS, Park MJ, Choi JE, Kim JY, Nam MS, Jeong YH (2007). Effects of Silk Protein Hydrolysates on Blood Glucose Level, Serum Insulin and Leptin Secretion in OLETF Rats. *J Kor Soc Food Sci Nutr*.

[12] Lee MS, Kim DM, Cho BN, Koo SJ, Jew SS, Jin DK, Lee SH (2003). Study on consequent body fat and serum lipid metabolism after cocoon hydrolysate, green tea leaves and dietary fiber supplementation. *J Korean Soc Chem Biotechnol*.

[13] Kim JS, Hwang HJ, Yun HY, Kim BK, Lee CH, Suh HJ, Lim KW (2013). Silk Peptide intake increases fat oxidation at rest in exercised mice. *J Nutr Sci Vitaminol*.

[14] Kim JS, Hwang HJ, Park JH, Yun HY, Suh HJ, Lim KW (2014). Silk peptide treatment can improve the exercise performance of mice. *J Int Soc Sports Nutr*.

[15] Lim KW, Kim JS, Jeon, YR, Hwang, HJ, Suh, HJ (2011). Measurement of resting metabolic rate using metabolic chamber in resting rats. *J Exerc Nutr Biochem*.

[16] Kim JS, Jeon YR, Hwang HJ, Suh HJ, Lim KW (2011). Effects of oral caffeine and capsaicin administration on energy expenditure and energy substrates utilization in resting rats. *J Exerc Nutr Biochem*.

[17] Park KJ, Hong SE, Do MS, Hyun CK (2002). Stimulation of insulin secretion by silk fibroin hydrolysate in streptozotocin-induced diabetic rats and db/db mice. *Kor J Pharmacogn*.

[18] Bonen A, Cambell S, Benton C, Chabowski A, Coort S, Han Z, Koonen D, Glatz J, Luiken J (2004). Regulation of fatty acid transport by fatty acid translocase/CD36. *proc Nutrition Soc*.

